# Prevalence of intestinal parasites among inmates in Midwest Brazil

**DOI:** 10.1371/journal.pone.0182248

**Published:** 2017-09-21

**Authors:** Larissa Gabrielle Curval, Adriana de Oliveira França, Henrique Jorge Fernandes, Rinaldo Pôncio Mendes, Lídia Raquel de Carvalho, Minoru German Higa, Eduardo de Castro Ferreira, Maria Elizabeth Cavalheiros Dorval

**Affiliations:** 1 Graduate Program in Infectious and Parasitic Diseases, Universidade Federal de Mato Grosso do Sul, Campo Grande, MS, Brazil; 2 Department of Animal Sciences, Universidade Estadual de Mato Grosso do Sul, Aquidauana, MS, Brazil; 3 Visiting Professor, School of Medicine, Universidade Federal de Mato Grosso do Sul, Campo Grande, MS, Brazil; 4 Department of Biostatistics, Biosciences Institute of Botucatu, Universidade Estadual Paulista, Botucatu, SP, Brazil; 5 Fundação Oswaldo Cruz, Campo Grande, MS, Brazil; Universita degli Studi di Parma, ITALY

## Abstract

**Background:**

Intestinal parasitic infections constitute a public health issue in developing countries, with prevalence rates as high as 90%, a figure set to escalate as the socioeconomic status of affected populations deteriorates. Investigating the occurrence of these infections among inmates is critical, since this group is more vulnerable to the spread of a number of infectious illnesses.

**Methods:**

This cross-sectional, analytical, quantitative study was conducted in July 2015 at prison facilities located in Midwest Brazil to estimate the prevalence of parasitic infection among inmates. For detection of parasites, 510 stool samples were examined by ether centrifugation and spontaneous sedimentation.

**Results:**

Eight parasitic species were detected, with an overall prevalence of 20.2% (103/510). *Giardia lamblia* and *Entamoeba histolytica*/*dispar* were the most frequent pathogenic parasites. *Endolimax nana* was the predominant non-pathogenic species. Nearly half of the subjects (53/103; 51.4%) were positive for mixed infection. Logistic regression revealed that inmates held in closed conditions were more likely to contract parasitic infections than those held in a semi-open regime (OR = 1.97; 95% CI = 1.19–3.25; *p* = 0.0085). A higher prevalence of parasitic infections was observed among individuals who had received no prophylactic antiparasitic treatment in previous years (OR = 10.2; 95% CI = 5.86–17.66; *p* < 0.001). The other factors investigated had no direct association with the presence of intestinal parasites.

**Conclusion:**

Infections caused by directly transmissible parasites were detected. Without adequate treatment and prophylactic guidance, inmates tend to remain indefinitely infected with intestinal parasites, whether while serving time in prison or after release.

## Background

Intestinal parasitic infections have long been a public health issue, particularly in developing countries, where more than two billion people are affected [1,2]. Social marginalization and lack of adequate medical care have increased the vulnerability of this population to other pathogenicities and morbidities associated with parasitic infections [3]. Furthermore, poor living conditions, lack of sanitation, and limited access to safe drinking water [4] have played an important role in the acquisition of these parasitoses, which become more frequent as socioeconomic conditions decline [5].

Globally, the number of confirmed cases is high. An estimated 1.5 billion individuals are infected with *Ascaris lumbricoides*, 1.3 billion with *Trichuris trichiura*, 1.05 billion with hookworms, 200 million with the *Entamoeba histolytica/dispar *complex, and 400 million with *Giardia duodenalis* [6,7]. Currently, *Ascaris lumbricoides* is the most prevalent parasitic infection, affecting roughly 30% of the population on the American continent [8]. Latin America and the Caribbean are home to some 210 million individuals who live below the poverty line and are severely affected by parasitic infections, owing to lack of effective sanitation [9,10].

In Brazil, intestinal parasitic infections have been exacerbated by flawed public health policies [11], as well as inappropriate socioeconomic and environmental approaches, which repercuss on the daily living conditions of many residents [12].

Socioeconomic and behavioral factors—including higher exposure to contaminants, poor standards of personal hygiene, malnutrition, mobility issues, psychological disorders, and stress—can make some population groups, such as prison inmates, more prone to parasitic infection [13,14]. Additionally, sedentary lifestyles and use of drugs, among other detrimental practices, can aggravate the recurrent health issues of inmates, such as respiratory, sexually transmissible, and parasitic illnesses [15]. Epidemiological analysis of these conditions has revealed a consistent link with the degree of insalubrity and lack of sanitation experienced by these individuals [16].

Data on the prevalence of intestinal parasitic infections within Brazilian prisons are scarce, and the few studies available involve small samples that are not sufficiently representative for analysis of parasitic diagnoses. In most prisons in underdeveloped countries, healthcare depends on underfunded systems that, because of poor structural planning and insufficient investment, have to rely on underqualified professionals.

This study investigated the prevalence of parasitic infections within prisons located in the Midwestern state of Mato Grosso do Sul to identify possible links between epidemiological factors and the emergence of intestinal parasitic infections in the incarcerated population. The study is part of an effort to map prevalence rates in all regions of the country and establish coordinated policies for the control of parasitic diseases, ultimately ensuring universal access to healthcare services and promoting sanitary and environmental education.

## Materials and methods

### Study type, location, and population

This cross-sectional, analytical, quantitative study was conducted in penal establishments in Campo Grande, the capital city of Mato Grosso do Sul. The inmates investigated were men and women aged 18 years or older serving sentences under closed conditions at the male-only Maximum Security Prison or the Women’s Prison, and men aged 18 years or older held under semi-open conditions at the Agricultural Penal Colony.

According to the Mato Grosso do Sul Agency for Penitentiary Administration (Agepen-MS), these facilities held 2097, 377, and 880 inmates at the time of the study, respectively. The 510-person sample investigated in this study comprised 240, 80, and 190 individuals, respectively.

Sample size was estimated from the formula $N=\frac{{\left(Z_{\propto }+Z_{\beta }\right)}^{2}∙2∙\bar{p}∙(1-\bar{p})}{{\left(\bar{d}\right)}^{2}}$, based on the following parameters: total number of inmates per prison, an estimated prevalence of parasitosis of 20% (± 5%), and a significance level of 5%. The technique employed was proportional stratified sampling for the number of individuals in each institution. Statistical treatment was performed with Epi Info 7.0 software.

Enrollment of volunteer participants and collection of stool samples were initiated in November 2014 and concluded in June 2015. Individuals with impaired capacity to exercise civil rights and fulfill civil duties or having impaired intellectual capacity were excluded, as were those for whom special types of consent are mandatory—e.g., members of Quilombola groups (residents of ethnically homogeneous, typically isolated rural communities of descendants of former Afro-Brazilian slaves) or indigenous communities.

### Sociodemographic, clinical, and epidemiological data

Interviews were conducted individually by trained healthcare professionals to ensure privacy, using a customized structured questionnaire (S1 Appendix A, S2 Appendix B.). Demographic and epidemiological data, as well as information on signs or symptoms suggestive of intestinal parasitic infection, were collected. The participants were informed about the subsequent collection of stool samples.

### Sample collection and testing

Stool samples were collected in universal specimen flasks containing MIF fixative, stored at room temperature, and processed at the Clinical Parasitology Laboratory of the Biological and Health Sciences Center at the Universidade Federal de Mato Grosso do Sul (UFMS). The centrifugation in ether [17] and spontaneous sedimentation [18] techniques employed are suitable for investigating structures of varying densities, such as protozoan cysts and helminth eggs and larvae. For logistic reasons, only one sample by inmate was collected. After that, two fecal sediment slides, obtained by each method and stained with Lugol solution, were examined.

### Data analysis

Potential risk factors typically associated with parasitic infection were taken into account for the analysis. These included type of prison facility, sanitation system, number of inmates per cell, age group, length of sentence, and behavioral aspects (habit of washing hands, previous antiparasitic treatment in the past two years, working in the vegetable garden, knowledge held on the meaning of parasitic infection, and having undergone previous stool tests). Logistic regression was used to investigate the effect of structural, social, and behavioral factors on the presence of parasitic diseases and of symptoms generally associated with these infections. Odds ratios (OR) and respective confidence intervals (CI 95%) were expressed for each level. Significance was set at *p* < 0.05. SAS 9.4 software was employed to analyze the data on pathogens and hosts. The McNemar test was used to compare dichotomous variables in the same sample when two parameters were present, whereas the Cochran test was employed for more than two parameters. Dichotomous variables in three independent samples were compared using Tukey’s test.

### Ethical considerations

The study design and data collection instrument were approved by the UFMS Ethics Committee for Research on Humans (permit 37800114.8.0000.0021). After receiving the test results and guidance on prevention of the diagnosed infections, the participants who tested positive for any pathogenic species were placed under specific clinical and therapeutic surveillance. Metronidazole and albendazole were prescribed for protozoans and helminths, respectively. Nutritional guidance was also provided.

## Results

As shown in Table 1, most subjects were male (84.3%) and from Mato Grosso do Sul (69.6%). Age ranged from 18 to 73 years. Most participants were single (63.1%). Skin color was self-reported mostly as brown (48%) or white (42.9%). Educational level was predominantly primary school (67.7%). Only a minority of individuals (2.5%) had studied to college degree level. Household income before imprisonment typically did not exceed three minimum wages.

**Table 1:** Sociodemographic characteristics of inmates serving sentences at three prison facilities in Mato Grosso do Sul, Midwest Brazil (*n* = 510).

Variables	*n* (%)
**Sex**	
Male	430 (84.3)
Female	80 (15.7)
**State of origin**	
Mato Grosso do Sul	355 (69.6)
Elsewhere in Brazil	155 (30.4)
**Age (years)**	
18–28	173 (33.9)
29–39	221 (43.3)
>39	116 (22.8)
**Marital status**	
Married	188 (36.9)
Single	322 (63.1)
**Skin color (self-reported)**	
Brown	245 (48.0)
White	219 (42.9)
Black	42 (8.2)
Yellow	4 (0.9)
**Educational level**	
Illiterate	10 (2.0)
Primary	345 (67.7)
Secondary	142 (27.8)
Tertiary	13 (2.5)
**Income before imprisonment (minimum wages)**	
0–1	143 (28.0)
1–2	153 (30.0)
2–3	128 (25.1)
≥4	86 (16.9)

*n* = number of participants

A total of 510 stool samples were examined. Overall positivity, defined as presence of one or more parasitic species, regardless of sample origin, was 20.2% (103 cases). Among positive samples, mixed infections were detected in 51.4% of cases. The remaining positive samples were single-species cases (Table 2). The *Endolimax nana/Iodamoeba bütschlii *complex accounted for most cases of mixed infection (31/103; 30.1%).

**Table 2:** Cases of single- and mixed-species intestinal infection among positively diagnosed inmates, by prison facility. Mato Grosso do Sul, Midwest Brazil (*n* = 103).

Species	Women’s Prison	Maximum Security Prison	Semi-open Colony	Total
	17/80	59/240	27/190	103/510
**Single-species infection (*n***; **%)**				
*Giardia lamblia*	0	6 (10.2)	4 (14.8)	10 (9.7)
*Entamoeba histolytica/dispar*	0	7 (11.9)	1 (3.7)	8 (7.8)
*Iodamoeba bütschlii*	0	2 (3.4)	0	2 (1.9)
*Entamoeba coli*	1(5.9)	7 (11.9)	3 (11.1)	11 (10.7)
*Endolimax nana*	0	11(18.6)	2 (7.4)	13 (12.6)
*Blastocystis* sp.	0	3 (5.1)	1 (3.7)	4 (3.9)
*Chilomastix mesnili*	1 (5.9)	0	0	1 (1.0)
*Taenia* sp.	0	1 (1.7)	0	1 (1.0)
	2 (11.7)	37 (62.7)	11 (40.7)	50 (48.6)
**Mixed infection (*n*; %)**				
*E*. *nana* + *I*. *bütschlii*	7 (41.1)	16 (27.1)	8 (29.6)	31 (30.1)
*E*. *nana* + *E*. *coli*	1 (5.9)	0	4 (14.8)	5 (4.8)
*E*. *coli* + *Blastocystis* sp.	1 (5.9)	0	0	1 (1.0)
*G*. *lamblia* + *E*. *coli*	1 (5.9)	0	0	1 (1.0)
*G*. *lamblia* + *I*. *bütschlii*	2 (11.8)	3 (5.1)	2 (7.4)	7 (6.7)
*I*. *bütschlii* + *Blastocystis* sp.	1 (5.9)	0	0	1 (1.0)
*E*. *nana* + *Blastocystis* sp.	1 (5.9)	0	1 (3.7)	2 (1.9)
*E*. *histolytica/dispar* + *E*. *coli*	0	1 (1.7)	0	1 (1.0)
*E*. *nana* + *I*. *bütschlii* + *E*. *coli*	1 (5.9)	0	0	1 (1.0)
*E*. *nana* + *G*. *lamblia*	0	0	1 (3.7)	1 (1.0)
*E*. *nana* + *E*. *histolytica/dispar*	0	2 (3.4)	0	2 (1.9)
	15 (88.2)	22 (37.3)	16 (59.3)	53 (51.4)

*Giardia lamblia* (19.4%) and *Entamoeba histolytica/dispar* (12.6%) were the most frequent pathogenic species, while *Taenia* sp. had the lowest prevalence (1.0%) (*p* < 0,05). Pathogenic parasites were more prevalent in the Maximum Security Prison (9.6%), followed by the Women’s Prison (7.5%) and the Semi-open Agricultural Colony (5.3%), but these differences were not significant (*p* > 0.05).

Of the non-pathogenic species, *Endolimax nana* (55.3%), *Iodamoeba bütschlii* (47.6%), and *Entamoeba coli* (27.2%) were the most frequent, while *Chilomastix mesnili* (1.0%) (*p* < 0.05) had the lowest prevalence. The prevalence of non-pathogenic parasites was similar in the two prisons operating under closed conditions (Maximum Security, 15.0%; Women’s, 13.8%) and significantly lower in the semi-open facility (8.9%) (*p* < 0.05). Table 3 shows the distribution of parasitic species in the 103 positive samples.

**Table 3:** Prevalence rates of intestinal parasites among inmates serving sentences at three prison facilities in Mato Grosso do Sul, Midwest Brazil (semi-open regime, *n* = 27; closed-regime Maximum Security, *n* = 59; closed-regime Women’s Prison, *n* = 17; total positive cases, *n* = 103).

Parasite	Semi-open Colony	Maximum Security Prison	Women’s Prison	*p*
**Pathogenic species (%)**				
*Giardia lamblia*	3.7	4.2	3.8	>0.05
*Entamoeba histolytica/dispar*	1.6^ab^	4.2^a^	0.0^b^	<0.05*
*Blastocystis* sp.	1.1	1.3	3.8	*>*0.05
*Taenia* sp.	0.0	0.4	0.0	>0.05
**Non-pathogenic species (%)**				
*Iodamoeba bütschlii*	5.3^b^	11.7^a^	13.8^a^	<0.05*
*Entamoeba coli*	4.2	5.4	8.8	>0.05
*Endolimax nana*	8.4^b^	12.9^ab^	20.0^a^	<0.05*
*Chilomastix mesnili*	0.0	0.0	1.3	>0.05

* Different letters (a,b) on the same row indicate significant differences (*p* < 0.05, Tukey’s test).

The effect of structural and behavioral factors on prevalence rates and OR values of parasitosis can be observed in Table 4.

**Table 4:** Logistic regression analysis of structural, social, and behavioral factors associated with prevalence of intestinal parasitic infection among inmates. Mato Grosso do Sul, Midwest Brazil.

Structural and social factors	Prevalence of parasitic infection(*n*; %)	Odds ratio* (95% CI^4^)	*p*
**Prison facility**			
WP^1^	17 (16.5)		0.031
MSP^2^	59 (57.3)		
SOC^3^	27 (26.2)		
WP *vs*. MSP		0.83 (0.45–1.53)	0.545
SOP *vs*. WP		1.63 (0.83–3.12)	0.156
MSP *vs*. SOC		1.97 (1.19–3.25)	0.008
**Sanitation**			
Sewage system	54 (52.4)	1.03 (0.67–1.59)	0.881
Cesspool	49 (47.6)		
**Inmates per cell**			
1–4	20 (19.4)		0.155
5–8	15 (14.6)		
9–12	32 (31.0)		
13–16	17 (16.5)		
>16	19 (18.5)		
**Age (years)**			
18–28	35 (34.0)		0.902
29–39	43 (41.7)		
>39	25 (24.3)		
**Time served**			
≤2 months	29 (28.2)		0.6323
>2 months to 2 years	40 (38.8)		
>2–9 years	32 (31.1)		
≥10 years	2 (1.9)		
**Habit of washing hands**			
Yes	97 (94.2)	1.74 (0.65–4.65)	0.271
No	6 (5.8)		
**Previous antiparasitic treatment**^**†**^			
Yes	18 (17.5)	10.18 (5.86–17.66)	<0.001
No	85 (82.5)		
**Works in vegetable garden**			
Yes	58 (56.3)	1.21 (0.78–1.85)	0.393
No	45 (43.7)		
**Knows meaning of term ‘parasite’**			
Yes	16 (15.5)	1.09 (0.60–1.98)	0.774
No	87 (84.5)		
**Previous stool test**^**†**^			
Yes	45 (43,7)	1.16 (0.75–1.71)	0.498
No	58 (56.3)		

^1^WP: Women’s Prison

^2^MSP: Maximum Security Prison

^3^SOC: Semi-open Colony

^4^CI: confidence interval

*Odds ratios were considered significantly different from 1.0 when *p* < 0.05.

^**†**^ Subjects were asked whether they had undergone a stool test or received prophylactic antiparasitic treatment in the previous two years.

Structural, social, and behavioral factors, when combined, correlated significantly with the probability (%) of intestinal parasitic infections in the prison facilities investigated (*p* = 0.031). Similar, but not significantly different, rates of parasitic infection were found at the Women’s Prison and Maximum Security Prison (OR = 0.83; 95% IC = 0.445–1.53; *p* = 0.55). By contrast, a highly significant difference (*p* = 0.008) in infection rates was observed between the Maximum Security Prison and Semi-open Colony (OR = 1.97; 95% IC = 1.19–3.25), where the likelihood of infection in the Maximum Security Prison, which operates under strictly closed conditions, was almost twice as high as in the semi-open facility.

Having received antiparasitic treatment in the previous two years was another factor influencing the occurrence of intestinal parasites (OR = 10.2; 95% IC = 5.86–17.66): untreated individuals were roughly ten times more likely to become infected that those given specific drugs (*p* < 0.001). The other structural, social, or behavioral aspects investigated did not prove significant.

The participants were asked about the presence of symptoms commonly associated with parasitic infection occurring at the time of interview or in the previous two years.

**Fig 1:**
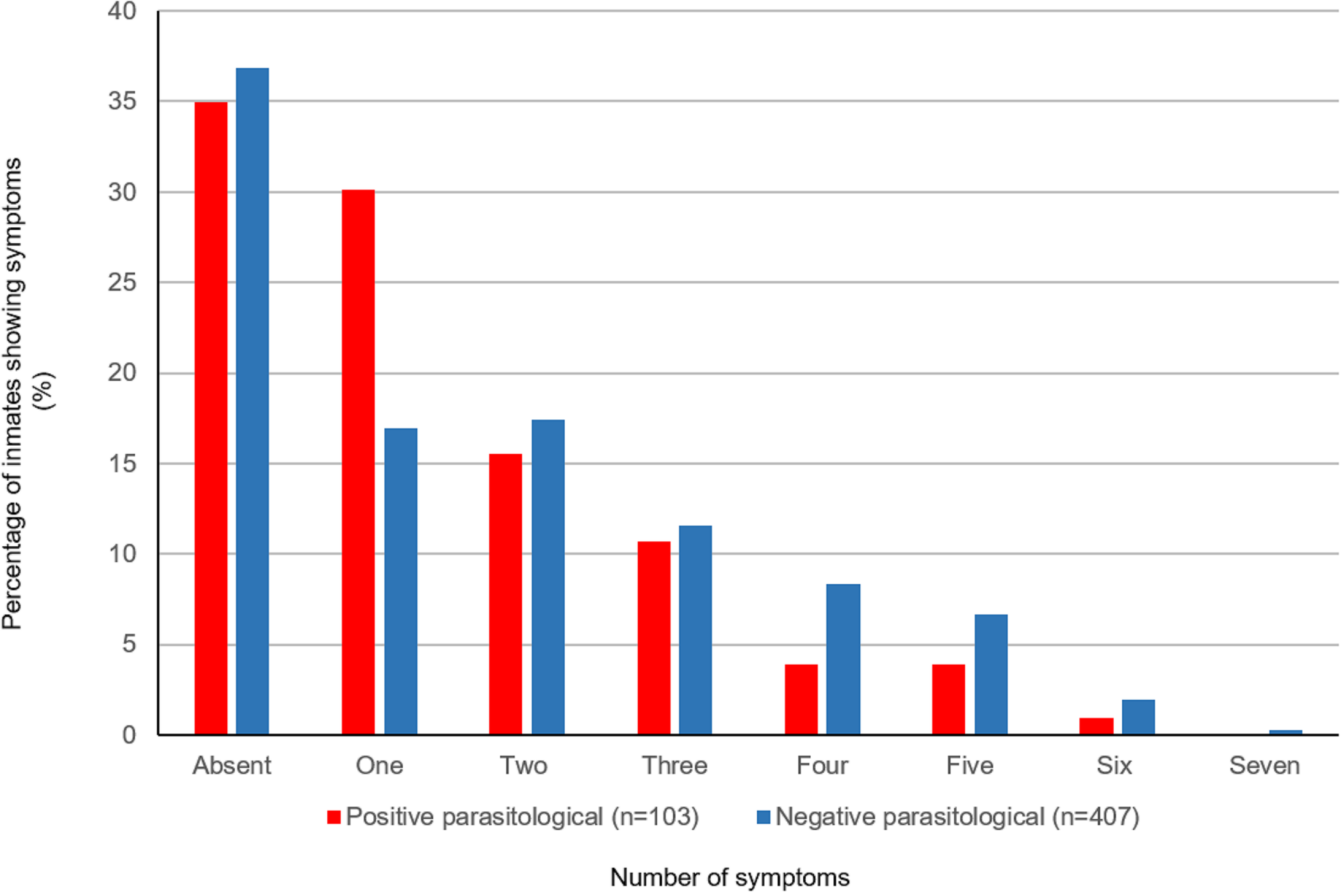
Number of symptoms experienced by group according to the results of parasitological examination. Symptoms presented by the studied population,distributed according to their number and percentage of patients with positive parasitological examination (red) or negative parasitological examination (blue).

Unspecific symptoms were the most commonly reported (Table 5), including abdominal pain, inappetence, and weight loss. The number of symptoms reported was similar for respondents with positive and negative parasitological findings, which demonstrates that the symptoms reported cannot be consistently associated with presence of parasites [Fig 1]. However, additional testing, employing more stool samples from the same individual, might have revealed correlations between parasitological results and reported symptoms.

**Table 5:** Symptoms reported by inmates with positive parasitological stool tests. Mato Grosso do Sul, Midwest Brazil (*n* = 103).

Symptoms	Prevalence (*n*; %)	Odds ratio*	*p*
**Diarrhea**			
Yes	15 (14.6)	1.12 (0.65–2.12)	0.560
No	88 (85.4)		
**Constipation**			
Yes	18 (17.5)	1.478 (0.84–2.58)	0.170
No	85 (82.5)		
**Anal itching**			
Yes	4 (3.9)	1.279 (0.43–3.84)	0.660
No	99 (96.1)		
**Abdominal pain**			
Yes	32 (31.1)	1.34 (0.84–2.13)	0.220
No	71 (68.9)		
**Inappetence**			
Yes	23 (22.3)	1.58 (0.95–2.63)	0.080
No	80 (77.7)		
**Weight loss**			
Yes	25 (24.3)	1.03 (0.62–1.71)	0.909
No	78 (75.7)		
**Sickness, nausea**			
Yes	13 (12.6)	1.54 (0.81–2.90)	0.183
No	90 (87.4)		
**Worm expulsion**^**†**^			
Yes	8 (7.8)	1.19 (0.53–2.64)	0.673
No	95 (92.2)		

*Odds ratios were considered significantly different from 1.0 when *p* < 0.05.

^**†**^ Subjects were asked about the presence or occasional expulsion of worms in the previous two years.

## Discussion

This is the first study on the incidence of intestinal parasitic infections among prisoners in Midwest Brazil.

Most participants were from the state where the prisons were located, although this distribution pattern is not typical of Brazilian prisons [19]. Most participants were male, single, young adults from low-income households, serving sentences under closed conditions of confinement. A small number had college degrees and were professionally stable. However, formal education did not exceed primary school level for 67.7%, implying low social and cultural attainment prior to incarceration, a pattern also reported elsewhere [20,21,22,23,24].

Studies on the prevalence of intestinal parasitic infections in Brazil remain scarce. Those studies available are generally fragmented and involve loosely defined population samples, such as public healthcare or daycare recipients, children attending public schools, or underprivileged communities [25]. A high prevalence of parasitic infections among young members from low-income households and with modest cultural and socioeconomic backgrounds has been reported for several areas in Brazil, with rates as high as 70–95% in the Amazonian region [5,12,26,27,28,29]. Overall, the most frequently detected parasites in the general population have been *Entamoeba coli*, *Giardia lamblia*, *Endolimax nana*, *Iodamoeba bütschlii*, *Chilomastix mesnili*, and *Entamoeba histolytica/dispar* [5,26,29], all of which were found in the present sample.

The 20.2% rate found for parasitic infection differs markedly from the 33.3% and 34% [20,21] rates reported for inmates in Southeast Brazil. These previous figures, however, are not amenable to comparison with the present study, as the samples involved were smaller and the diagnostic methods limited. Much higher prevalence rates were found among inmates in Honduras, Ethiopia, and Nigeria, with infections by one or more species in 61.8–77% of prisoners [24,30,31,32], while infections may have been acquired before incarceration. Another factor precluding direct comparisons was that only one stool sample was examined for each subject in the present study, either because participants refused retesting or owing to difficulties revisiting the same prisons. Repeat testing might have revealed higher prevalence rates.

Despite the higher frequency of non-pathogenic species, the socioeconomic and sanitary conditions experienced by the subjects should not be overlooked, as these conditions are conducive to the spread of pathogenic agents [33,34].

The most prevalent pathogenic species were *Giardia lamblia* and *Entamoeaba histolytica/dispar*. Among the non-pathogenic parasites, *Endolimax nana* and *lodamoeba bütschlii* predominated. However, almost 50% of cases had mixed infection, which has been reported at lower rates for residents of uncrowded environments [5,28,29]. Giardiasis represents a public health concern, not only for its association with increased morbidity and medical expenditures, but also for the emergence of resistant strains [35,36]. Under humid conditions, *Giardia lamblia* cysts can remain viable for several months. In addition, the cysts can be resistant to hypochlorite [37]. Infection with *Giardia lamblia* may occur at much higher rates than reported, given that cysts are intermittently expelled in stools.

In a study investigating a small group of inmates in São Paulo state, *Giardia lamblia* (9.68%) and *Endolimax nana* (29.03%) infections predominated [20], albeit at lower rates than in the present sample, where infection with protozoans may also be ascribed to person-to-person transmission, since these microorganisms are often found in overcrowded environments, a feature that underscores the importance of implementing prophylactic guidelines on the physical environment and personal hygiene [20,38]. All the penal institutions investigated in the present study rely on chemical treatment of water to control infectious agents.

Soil-transmitted helminths are largely associated with low-quality water or with the habit of eating raw vegetables [39,40,41], factors absent from the prisons investigated. Concerning food-borne helminthic infection, although only a single case of taeniasis was diagnosed, this finding raises concerns over the possibility of acquiring neurocysticercosis, the principal cause of epilepsy in developing countries, a more serious forms of Taenia solium infection. [42]. Direct contact with contaminated soil was not observed in any of the locations investigated, including the Semi-open Agricultural Penal Colony. In the present study, material difficulties prevented the use of specific techniques for detection of helminth species such as *Enterobius vermicularis* and *Strongyloides stercoralis*, a fact which may have skewed the results.

Overcrowding, lack of preventive, prophylactic measures, and scant attention to primary healthcare are common features inherent to the majority of prisons located in Brazil and poorer countries [19,32]. In the present sample, for instance, 30 or more inmates shared the same cell and sanitary facilities, although overcrowding alone may not account for environmental contamination or spread of parasitic diseases, since no statistical significance was observed for the number of inmates per cell.

Although parasitological surveys in other Brazilian prisons did not include data on educational level [20,21], the potential for parasite spread has been reported as exhibiting inter- and intraregional variability, being shaped by sanitary, educational, and economic conditions, as well as by the agglomeration index of a given population and levels of soil, water, and food contamination [11,43]. In the present study, however, no direct link with these factors was observed, despite the poor educational background and low household income of most subjects. Most of the participants (83.5%) held no knowledge on intestinal parasitic infections, were unaware of the utility of parasitological exams, and had only been tested in their childhood or adolescence. Age group, however, bore no relation with the prevalence of parasitism (minimum age in the sample was 18 years), in contrast with studies showing that prevalence rates differ between adolescents and adults [22,23,24,44].

No significant differences were found for the association between infection and length of incarceration. Inmates who had been serving for less than two months accounted for almost 30% of positive cases, while those serving for more than 10 years represented only 2% of infected subjects, suggesting previous infection should not be ruled out.

None of the self-reported symptoms were significantly associated with parasitic infection, suggesting that diagnosis cannot be exclusively based on clinical signs. Although complaints of diarrhea did not correlate with confirmed cases of infection with *Entamoeba histolytica*/*dispar*, *Giardia lamblia*, or *Blastocystis* sp., inmates presenting with this symptom may be chronic carriers of these parasites.

Taken together, the structural, social, and behavioral aspects investigated revealed the odds ratios of infection to be twice as high in the Maximum Security Prison than in the Semi-open Colony. Overall, presence of parasitic infection was influenced by antiparasitic treatment received in the previous two years, which corroborates findings of a recent study [24] and highlights the importance of prophylactic empirical treatment. Factors predisposing inmates to increased vulnerability to parasites are related to confinement conditions and the absence of measures to raise awareness on hygiene and sanitary practices [45]. Once infected, individuals tend to disseminate parasites for long periods, unless effectively treated. Poor hygiene and sanitary conditions create a vicious cycle that may explain the recurrence of intestinal infection in prisons, and also reflects a lack of awareness of basic notions of hygiene in the general population [46,47]. In the absence of prophylactic measures and timely diagnosis, the health status of inmates can be worse at release than at admission [48].

Prisoners are entitled to all the fundamental rights of human beings, including the right to the highest standards of physical and mental health [19]. The Brazilian Plan for Surveillance and Control of Enteroparasitosis [49] relies on parasitosis prevalence, morbidity, and mortality data to generate analytical studies that will guide the design of strategies for the control of intestinal parasitic infections. The plan, therefore, should be implemented in all prison facilities in the country.

## Conclusions

This parasitological survey, the first of its kind conducted in prisons in Midwest Brazil, investigated pathogenic and non-pathogenic intestinal parasites typically associated with poor hygiene and sanitary conditions. Clinical symptoms consistent with intestinal parasitic diseases were observed, although the presence of symptoms was not correlated with the incidence of parasitic infections. Intestinal parasites were more frequent in prisons operating under closed conditions, which suggests that overcrowding is a factor promoting the development of infection. Inmates who reported previous treatment for established infection or prophylactic treatment proved to be less vulnerable to parasitosis.

The findings demonstrate that difficulties performing individual tests for accurate diagnosis among hard-to-reach populations, as is the case with inmates, can be at least partly circumvented by empirical prophylactic treatment of intestinal parasitosis.

The living conditions of inmates in the prisons investigated draw attention to the need for improved primary care, including effective measures to prevent and control intestinal parasitic infections, particularly those caused by pathogens transmitted from person to person.

## Supporting information

S1 Appendix Form of consent (FC). (PDF)

S2 Appendix Form to obtain data. (PDF)

S1 Data available Species. (XLSB)

S2 Data available Inmates. (XLSB)
