# Supplementary material for: Prevalence of intestinal parasites among inmates in Midwest Brazil
Source: PLoS One. 2017 Sep 21;12(9):e0182248. doi: 10.1371/journal.pone.0182248 (PMC5608187; doi:10.1371/journal.pone.0182248)
Supplement: S1 Appendix — (PDF) [file pone.0182248.s001.pdf]

Serviço Público Federal
Ministério da Educação
Fundação Universidade Federal de Mato Grosso do Sul

APPENDIX A

FORM OF CONSENT (FC) – "THE STUDY OF THE PREVALENCE OF PARASITIC INFECTIONS AMONG INMATES OF THE PRISONAL SYSTEM OF THE MID-WEST REGION, BRAZIL".

You are being invited to participate as a volunteer in a research. After being aware of the following information in case of accepting to take part in the study you have to sign in the end of this document, which has a copy that you may keep it with. One of them is yours and the other remains with the principal researcher. In case of refusal you will not be penalized in any way. If in doubt you can search the Ethics Committee of the Federal University of Mato Grosso do Sul by this phone number: +5567 33457186.

Intestinal parasitic diseases affect a large portion of the population, especially the ones living in vulnerable conditions. The aim of this study is to identify the prevalence of intestinal parasitic infections in the prison population of the city of Campo Grande, located in the Mid-West region - Brazil. For this main reason, all these groups of prisoners are being invited to participate in this study.

Your participation in this research will consist in answering a questionnaire/form and collect a stool sample to conduct parasitological examinations. Its participation will not entail any risk to your health. Your participation in this survey is totally voluntary, free, unpaid, and you can withdraw your consent at any time without any harm to yourself. By participating in this survey you will receive some information about parasitic diseases, will receive a copy of the result of its examination, in addition to the appropriate medical treatment. The delivery of the results will be carried out by qualified researchers to clarify all your doubts about the treatment.

What are supposed to happen to your stool sample collected in this study? It will be stored and processed safely in the Laboratory of Clinical Parasitology at Federal University of Mato Grosso do Sul, under the responsibility of the PhD Professor Maria Elizabeth Cavalheiros Dorval and of the Master Degree student Larissa Gabrielle Curval. After diagnosis if you do not agree with the storage of your faeces, the same shall be disregarded and thrown away.

The record of your participation in this survey will be kept confidential, however, only the Ethics Committee and researchers can have access to the information obtained. Reports and or publications in scientific journals and events that result from this research are strictly prohibited to identify your person/name.

Your participation in the study is voluntary. You can choose not to take part in this study, or may withdraw at any time. You will not lose any benefits to which you are entitled.

To questions or problems related to this study, you can call to Professor Maria Elizabeth Cavalheiros Dorval, on phone number +556733457691. To questions about your rights as a participant in this study you can call the Ethics Committee in Research with Human Beings of the Federal University of Mato Grosso do Sul, Number: +556733457186.

I declare that I understood the objectives, risks and benefits of my participation in the research, and I agree to participate. If you are not able to read, a person will be reading this Term of Consent to you at this time and if you agree with what you are hearing, you may put your fingerprint on this document.

You will receive a copy of this term which appears the institutional phone responsible researcher (+556733457691) and the Research Ethics Committee (+556733457186) where you can ask questions about this research project and about your participation, now or at any time you wish.

Participant's full name:..

Participant's signature:..

Researcher's signature:..

Place and Date: ....
