# Supplementary material for: Prevalence of intestinal parasites among inmates in Midwest Brazil
Source: PLoS One. 2017 Sep 21;12(9):e0182248. doi: 10.1371/journal.pone.0182248 (PMC5608187; doi:10.1371/journal.pone.0182248)
Supplement: S2 Appendix — (PDF) [file pone.0182248.s002.pdf]

Nº...............

APPENDIX B

FORM TO OBTAIN SOCIOECONOMIC, DEMOGRAPHIC AND EPIDEMIOLOGICAL DATA – RESEARCH PROJECT "PREVALENCE OF PARASITIC INFECTIONS IN INMATES OF THE PRISONAL SYSTEM OF MID-WEST REGION, BRAZIL".

PRISON UNIT: ....

Gallery:..Number of inmates:...

Size of the cell?... The cell has windows?............................ How many?.........

The institution has potable water? ( ) Yes ( ) No

In case of negative answer, how it is obtained?...

The institution has sewerage system? ( ) Yes ( ) No

In case of negative answer, how it is eliminated? ....

1. Name: ....

2. City of origin:... 3. State of origin:............... 4.Birth Date: ..../......./........

5. Age: .... 6. Nationality: .... 7. Gender: .... 8. Marital status :................................

9. Prison time?.. 10. Cell number:..

11. How many people sharing the same cell :..

12. Ethnicity: a) Caucasian b) Black c) Brown d) Asian

13. What is your Education Level?

Illiterate (a) ( )

Incomplete Primary ( )

Complete Primary ( )

Incomplete Second ( )

Complete Second ( )

Incomplete Higher ( )

Complete Higher ( )

Technical Course ( )

14. Before being arrested what kind of job did you use to have?...

15. Have you dealt with vegetable Garden? ( ) Yes ( ) No

16. Adding the income of all persons who live with you in the same household (including your income before being arrested), which were your family's monthly income?

( ) until 1 Minimum Wage ( ) 2 to 3 Minimum Wage

( ) 1 to 2 Minimum Wage ( ) Above 4 Minimum Wage

17. House type: ( ) Brick ( ) Wood ( ) Shed Other type: ___________________________

18. How many rooms in your house?... How many bathrooms? ....

19. What type of sanitation system in your house? ( ) Sewage ( ) Cesspool

20. Do you house has access to sewerage system? ( ) Yes ( ) No Electrical Energy? ( ) Yes ( ) No

Potable Water? ( ) Yes ( ) No

21. Do you know what parasites mean? ( ) Yes ( ) No

22. Do you know the way of transmission of parasites? ( ) Yes ( ) No

....

23. Do you have or have ever had any parasitic disease in the previous 2 years? ( ) Yes ( ) No ( ) Do not know.

How long:................

24. Have you undergone any anti-parasitic treatment in the previous 2 years? ( ) Yes ( ) No ( ) Do not know.

How long :...............

25. Do you use to wash hands before eating? ( ) Yes ( ) No

26. Do you use to wash hands after going to the bathroom? ( ) Yes ( ) No

27. Have you ever done a stool exam in the previous 2 years? ( ) Yes ( ) No

How long:................

28. Do you have any sympthoms?

		Frequency?
Diarrhea	( ) Yes ( ) No	
Constipation	( ) Yes ( ) No	
Anal itching	( ) Yes ( ) No	
Abdominal pain	( ) Yes ( ) No	
Lack of appetite	( ) Yes ( ) No	
Weight loss	( ) Yes ( ) No	
Sickness and Nausea	( ) Yes ( ) No	
Elimination of worms	( ) Yes ( ) No	
